# CD10 expression in urinary bladder urothelial carcinoma is associated with high-tumor grade and stage

**DOI:** 10.12669/pjms.39.1.6268

**Published:** 2023

**Authors:** Jaudah Al-Maghrabi

**Affiliations:** Jaudah Al-Maghrabi Department of Pathology, Faculty of Medicine, King Abdulaziz University, Jeddah, Saudi Arabia Department of Pathology, King Faisal Specialist Hospital and Research Centre, Jeddah, Saudi Arabia

**Keywords:** CD10 immunoexpression, Urinary Bladder Carcinoma, Grade Stage Survival

## Abstract

**Objectives:**

Primary urinary bladder carcinoma is a common cancer worldwide. There is limited published data about CD10 immunoexpression pattern in urothelial bladder carcinoma (UBC). This study aims to examine CD10 immunoexpression in UBC and evaluate its relationship with clinicopathological parameters.

**Methods:**

The retrospective study examined 130 samples of UBC tissue and 30 samples of non-neoplastic urothelial bladder tissue, which were obtained from the Anatomic Pathology Department, King Abdulaziz University, Jeddah, Saudi Arabia. The project started in June 2019 and completed in February 2021. Tissue microarrays (TMA) were prepared from paraffin blocks and tissue sections prepared from the recipient blocks were used for immunohistochemistry studies utilizing CD10 antibody. The immunostaining results were recorded and analyzed.

**Results:**

Positive staining of CD10 was observed in 64 (49%) cases of UBC and was not detected in any non-neoplastic urothelium samples. CD10-positive staining was identified in 36.7% and 66.7% of low and high-grade tumors, respectively. There was an association between positive CD10 immunostaining and high tumor grade (*p*=0.006) and muscularis propria invasion (p=0.007). There was no association between CD10 immunoexpression and age, gender, nodal and distant metastasis, lymphovascular invasion, and tumor recurrence. CD10 immunoexpression was not associated with the probabilities of overall survival (log rank 1.663, p=0.197) or disease-free survival (log rank 1.637, p=0.201).

**Conclusions:**

In UBC, CD10 immunoexpression is associated with higher tumor grade and muscle invasion, but it is not associated with patient survival or other clinicopathological parameters. CD10 immunoexpression cannot be used as a biomarker for poor prognosis in UBC.

## INTRODUCTION

Urothelial bladder carcinoma (UBC), also known as Transitional cell carcinoma (TCC), is the most common primary urinary bladder carcinoma. In Saudi Arabia, UBC represents 3.8% of cancers in males.^1^ There is limited published data about the relationship between CD10 immunoexpression and clinicopathological parameters in UBC patients. CD10 has been reported to be associated with tumor grade and stage in urothelial carcinoma and to be a sign of poor prognosis. However, the relationship between CD10 immunoexpression and patient survival is very limited and has only been evaluated in three previous studies.^2^-^4^ There have been no studies in Saudi Arabia regarding the pattern of CD10 immunoexpression in bladder cancer. Therefore, this study evaluates the relationship between CD immunoexpression and clinicopathological parameters, including patient survival, in a relatively large sample of urothelial carcinomas.

## METHODS

A total of 130 bladder urothelial carcinomas specimens were investigated and examined by immunohistochemical staining along with 30 normal urothelial tissue samples. The patients’ clinical data and pathological blocks were collected from the Anatomic Pathology Department at King Abdulaziz University Hospital, Jeddah, Saudi Arabia. Tumor grades were reviewed and reclassified according to the World Health Organization’s classification as low- or high-grade urothelial carcinomas.^5^ Stages of tumors were reviewed and classified according the cancer staging atlas of the American Joint Committee On Cancer.^6^ A summary of clinicopathological data is shown in Table-I. Approval of the TMA study was obtained from the Institutional Biomedical Research Ethics Committee (Reference No 1127-13). The process of the was in accordance with the revised Declaration of Helsinki, 2000. As routine at our institution, an informed written consent was obtained from each patient to obtain permission to utilize their pathological tissue specimens for laboratory studies. The project started in June 2019 and completed in February 2021.

**Table-I:** Clinicopathological parameters of tumors (*n*=130).

Parameter		Number (%)	CD10 Immunoexpression		P value

			Negative	Positive	
Gender	Male	109 (83.4%)	58 (53.2%)	51(46.7)	0.233*
	Female	21 (16.2%)	8 (38.1%)	13 (61.9%)	
Age	< 60 years	55 (42.3%)	29 (52.7%)	26 (47.3%	0.594*
	≥ 60 years	75 (57.7%)	37 (49.3%)	38 (50.7%)	
Grade	Low grade	60 (46.2%)	38 (63.3%)	22 (36.7%)	0.006*
	High grade	70 (53.8%)	28 (44.4%)	42 (66.7%)	
Muscle invasion	Negative	63 (48.5%)	40 (63.5%)	23 (36.5%)	0.007*
	Positive	60 (46.1%)	23 (38.3%)	37 (61.7%)	
	Unknown	7 (5.4%)	3 (42.9%)	4 (57.1%)	
Nodal metastasis	Negative	112 (86.2%)	59 (52.7%)	53 (47.3%)	0.202*
	Positive	12 (9.2%)	4 (33.3%)	8 (66.7%)	
	Unknown	6 (4.6)	3 (50%)	3 (50%)	
Distant metastasis	Negative	123 (87.7%)	62 (50.4%)	61 (49.6%)	0.698*
	Positive	7 (7.7%)	4 (57.1%)	3 (42.9%)	
Lymphovascular invasion	Negative	114 (87.7%)	60 (52.6%)	54 (47.4)	0.169*
	Positive	10 (7.7%)	3 (30%)	7 (70%)	
	Unknown	6 (4.6)	3 (50%)	3 (50%)	
Local disease recurrence	Negative	85 (65.4%)	49 (57.6%)	36 (42.4%)	0.191*
	Positive	45 (34.6%)	20 (44.4%)	25 (55.6)	

* Chi-Square Test.

### Tissue Microarray:

The tissue microarray (TMA) was prepared as described.^7^,^8^ Histopathology slides of primary UBC and of non-neoplastic urothelial bladder tissue were reviewed and selected areas were marked. Areas that showed poor cellular preservation, necrosis, autolysis, tissue artefacts, and the area that contained predominant stromal tissue were avoided. Areas of the donor paraffin blocks that correspond to the selected sections were used to obtain two cores of neoplastic and non-neoplastic tissue. The cores were then inserted into recipient blocks using TMA machine (TMA Master 1.14 SP3 from 3D Histech Ltd., Budapest, Hungary). Sections from the recipient TMA blocks with a 4-µm-thickness were used for immunohistochemistry studies.

### Immunohistochemistry:

Constructed TMAs of paraffin-embedded tissue blocks were cut and mounted on slides positively charged (Leica Microsystems Plus Slides). Immunohistochemistry was done utilizing an automated immunostainer (Benchmark ULTRA, Ventana® Medical Systems Inc., Tucson, AZ, USA) with a mouse monoclonal antibody to CD10 (clone 56C6; dilution 1:10; CELL MARQUE). Basically, 4-μm sections from formalin-fixed paraffin-embedded specimens were cut on Poly-L-lysine coated slides. The sections then deparaffinized, dehydrated and were incubated with primary antibody at 1:10. Counterstaining with hematoxylin was done, then rinsed and mounted. Membrane and/or cytoplasmic staining was evaluated. Positive control slides of a reactive lymph node tissue known to be CD10 positive were used. Negative control (substitution of the primary antibody with Tris-buffered saline) was utilized.

### Interpretation of CD10 Immunostaining:

CD10 immunoexpression were divided as follows: Score-0 (negative staining) was given when <5% cells were positive; Score-1 (positive), 5%–50% cells positive and Score-2 when >50% positive cells were present. Tissue positive staining in at least 5% of targeted cells (Scores 1 and 2) was regarded as “CD10-positive”. The 5% cut off value was used as previously described.^9^ Tissues with no or less than 5% staining of targeted cells (score 0) were regarded as “CD10-negative”.

### Statistical analysis:

The chi-squared test and the Fisher exact test were used to assess the differences between two groups of variables. Kaplan-Meier method was used to evaluate the overall survival (OS) and disease-free survival (DFS) with the log-rank (Mantel-Cox) comparison test. DFS was calculated as the time from pathological diagnosis to the time of recurrence (or date of the last visit with disease-free status). SPSS® (IMB, NY, USA) software package, version 20 was used for statistical analyses. P <0.05 was considered statistically significant.

## RESULTS

**Fig.1:**
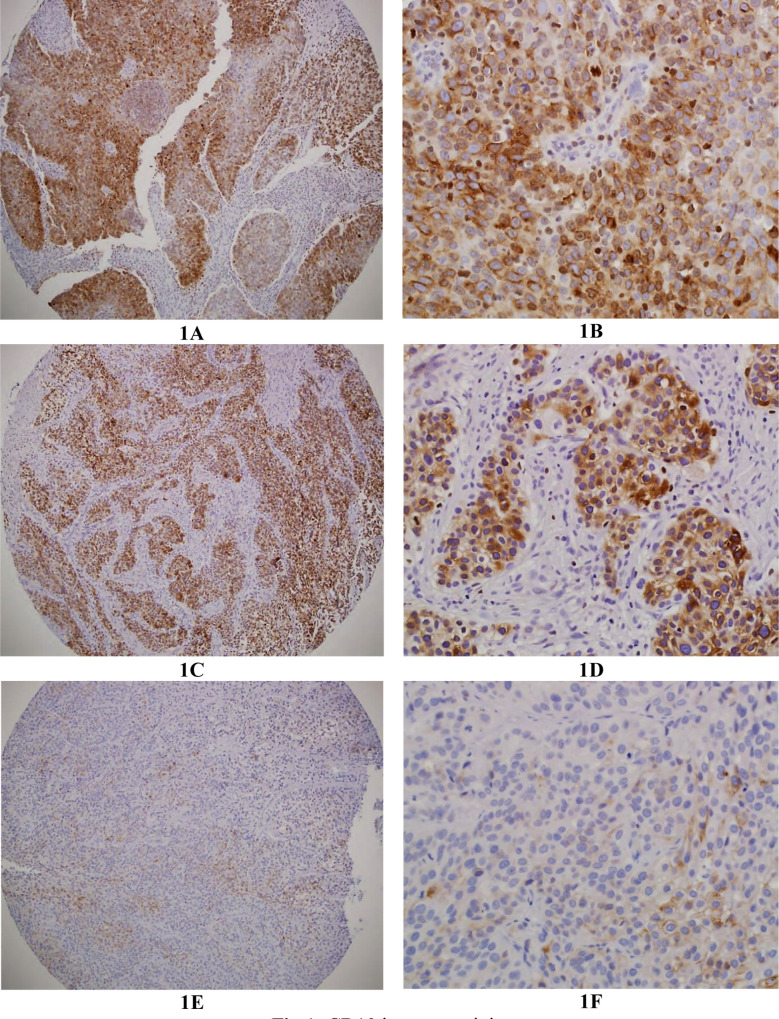
CD10 immunostaining. (A) Section from high grade urothelial bladder carcinoma showing strong positive immunostaining (X100). (B) High power (X400). (C) Section from low grade urothelial bladder carcinoma showing strong positive immunostaining (X100). (D) High power(X200). (E) Section from low grade urothelial bladder carcinoma showing focal weak CD10 immunostaining(X100). (F) High power(X100).

**Fig.2:**
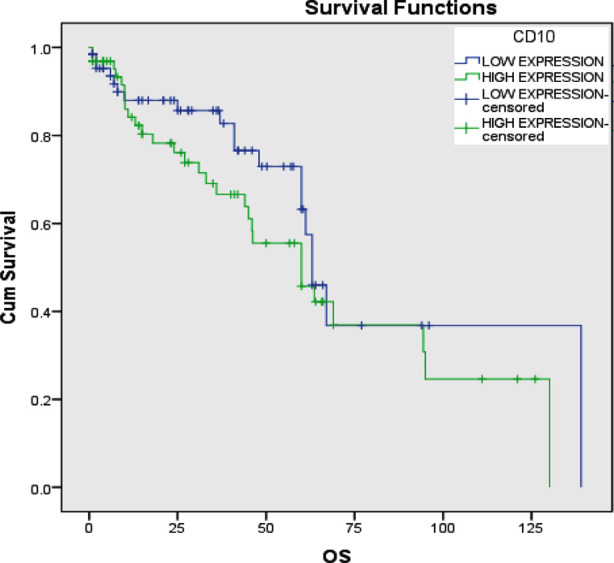
Overall survival curve (Kaplan Meier) in relation to CD10 immunoexpression in UBC patients. There is no association between CD10 immunostaining and OS (log rank 1.663, p=0.197).

**Fig.3:**
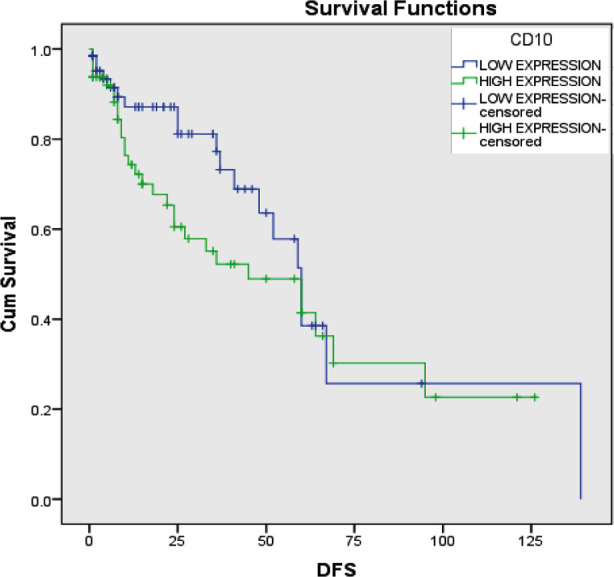
Disease-free survival curve (Kaplan Meier) in relation to CD10 immunoexpression in UBC patients. There is no association between CD10 immunostaining and DFS (log rank 1.637, p=0.201).

The clinicopathological features of the cases are summarized in Table-I. Among the 130 cases, 109 were male (88.9%), and 21 (11.1%) were female. All 130 cases of bladder carcinomas were low- or high-grade urothelial type. There were 60 cases of low-grade urothelial carcinoma (46.2%) and 70 cases with high grade urothelial carcinoma (53.8%). Positive CD10 staining was observed in 64 (49%) cases of UBC and was not detected in any non-neoplastic urothelium (Fig.1 and Table-I). The immunoexpression pattern of CD10 in UBC and non-neoplastic urothelium is summarized in Table 2. There was an association between positive CD10 immunostaining and high tumor grade (*p*=0.006) and muscularis propria invasion (p=0.007), which reflect tumor stage. CD10-positive staining was identified in 36.7% and 66.7% of low and high grade tumors, respectively and in 36.5% and 61.7% of non-muscle-invasive Bladder carcinomas, T1aT1 and muscle-invasive bladder carcinoma, T2-T4. There was no association between CD10 immunoexpression and age, gender, nodal and distant metastasis, lymphovascular invasion, or tumor recurrence (Table-II). CD10 immunoexpression was not associated with the probabilities of overall survival (log rank 1.663, p=0.197) or disease-free survival (log rank 1.637, p=0.201) (Fig.2 and 3).

**Table-II:** Categories of CD10 immunohistochemistry staining in bladder urothelial carcinoma and non-neoplastic urothelial tissue.

	Primary tumor (n=130)	Normal urothelium (n=30)	p value
CD10 negative	66 (51%)	30 (100%)	0.00001
CD10 positive	64 (49%)	0 (0%)	

* The Fisher exact test statistic value is 0.00001. The result is not significant at p < 0.05.

## DISCUSSION

CD10 is a membrane-associated peptidase that is related to peptide cleavage and is believed to be associated with neoplastic progression. The function of CD10 is to inactivate a number of signaling peptides. Physiologically, CD10 is highly expressed in epithelial cells of the lungs, kidneys, breast, placenta, and others. In this study, CD10 was only expressed in urothelial carcinomas and was not detected in normal urothelial mucosa. There was a strong association between CD10 immunoexpression and tumor grade and muscle invasion (but not with other clinicopathological parameters, including age, gender, nodal and distant metastasis, lymphovascular invasion, and tumor recurrence. There was no association between CD10 immunoexpression and OS or DFS.

Many studies have identified a difference in CD10 immunoexpression in tumors and non-neoplastic epithelial mucosa in different human cancers. In UBC, the results are controversial. CD10 was demonstrated to be associated with higher tumor grade in UBC,^10^-^12^ as well as higher tumor stage^10^ and lymph node metastasis.^2^ Some studies did not find an association between CD10 immunoexpression and tumor stage,^11^ tumor grade,^2^, muscle invasion^2^, lymph node metastasis,^2^ or vascular space invasion.^2^ In contrast, Bircan et al.^13^ found that CD10 was inversely correlated with tumor invasiveness and pathological stage.

The relation between CD10 immunoexpression and survival in patients with UBC has only been evaluated in three previous studies^2^-^4^ and was found to be associated with shorter OS. In the present study, CD10 immunoexpression was evaluated in a relatively large number of transitional-type UBC cases to check the relation between CD10 immunoexpression and patients’ survival. The results did not reveal any significant association between CD immunoexpression and OS or DFS, which contradicts other studies.^2^-^4^ A limitation in two of these previous reports is the relatively small number of patients included. Further studies with more patients and longer periods of follow-up will probably shed lighter on this relationship.

The present study and most of the previous studies evaluated CD10 immunoexpression in urothelium and not stromal cells. In the present study, no significant stromal immunoexpression was noticed. Some studies evaluated stromal CD10 immunoexpression and found no relation with tumor grade, stage, presence of lymph node or distant metastases, or survival.^2^ In a study that compared CD10 immunoexpression in the urothelial tissues and stroma, high tumorous CD10 was associated with tumor stage and grade, while stromal CD10 was associated with younger age.^14^ The contradiction regarding stromal immunoexpression is probably related to the different clones. Many studies revealed a relation between CD10 immunoexpression and apoptosis.^15^ In the current study, none of the normal or reactive urothelial tissues revealed CD10 immunoexpression. The lack of immunoexpression in normal urothelial tissues indicates a possible relation between CD10 immunoexpression and urothelial malignant transformation. No previous study on CD10 immunoexpression in bladder cancer has been publish in Saudi population.

CD10 has been shown to be associated with tumor progression in some tumors, like melanoma.^16^ Epithelial immunoexpression of CD10 was found to be associated with tumor grade, stage, and lymph node invasion in colorectal cancer.^17^ On the other hand, the stromal immunoexpression of CD10 was found to be associated with a larger tumor size in colorectal carcinoma,^18^ lymph node metastasis, recurrence and disease-free in breast carcinoma,^19^-^21^ and gastric carcinoma.^22^

### Limitations of the study:

This study utilized tissue microarray material, which includes representative cores of tissue. Normal or dysplastic mucosa adjacent to the tumor were not evaluated for CD10 immunoexpression. This is considered a limitation of the study.

## CONCLUSION

In conclusion, although CD10 is associated with higher tumor grade and stage, it is not associated with patient survival and cannot be used as a biomarker of poor prognosis in UBC. Further studies with a larger sample and longer follow-up period are recommended to explore the prognostic significance of CD10 in urothelial carcinoma further.
